# The Changing Incidence and Prevalence of Falls and Its Disability Burden Among the Geriatric Population in Saudi Arabia from 1990 to 2019: A Longitudinal Analysis Using Global Burden of Disease Study Data

**DOI:** 10.7759/cureus.49117

**Published:** 2023-11-20

**Authors:** Saad M Bindawas

**Affiliations:** 1 Rehabilitation Sciences, King Saud University, Riyadh, SAU; 2 Disability Research, King Salman Center for Disability Research, Riyadh, SAU

**Keywords:** saudi arabia, fall prevention, disability burden, prevalence, incidence, gender disparities, older adults, falls

## Abstract

Background: Falls represent a significant and growing public health issue among older adults worldwide. This study provides a comprehensive analysis of the trends in the incidence, prevalence, and disability burden of falls among older adults in Saudi Arabia over 29 years, with a focus on gender disparities.

Methods: Utilizing the Global Burden of Disease (GBD) Study data, this observational analysis tracked the epidemiology of falls from 1990 to 2019. The study employed ICD-9 and ICD-10 codes to identify falls, analyzing incidence, prevalence, disability-adjusted life years (DALYs), and years lived with disability (YLDs), stratified by gender and reported with 95% uncertainty intervals (UIs).

Results: The incidence and prevalence of falls increased for both genders from 1990 to 2019, with males showing a higher relative increase in prevalence rates (57% for males vs. 26% for females). The disability burden, indicated by DALYs, increased by 4% for males and decreased by 10% for females, whereas YLDs saw an increase of 38% for males and 8% for females. The analysis highlights a notable rise in both the frequency of falls and their associated disability, with gender-specific variations emphasizing greater impacts on males.

Conclusions: The findings illustrate a significant increase in fall-related incidents and associated disabilities among older adults in Saudi Arabia, with distinct gender differences. These trends call for targeted public health interventions and further research into the underlying causes of falls, risk factors, and effective prevention strategies. Such measures are essential to mitigate the impact of falls, improve health outcomes, and enhance the quality of life for the aging population.

## Introduction

Falls among older adults represent a significant global public health concern, leading to disability, morbidity, and mortality [1-3]. As the leading cause of injury-related death in adults 60 and older, falls, for example, contributed to over 38,000 fatalities in 2020 in the United States [4]. Worldwide, 684,000 fatal falls occur annually, ranking second to road traffic injuries among unintentional injury-related deaths [5].

Falls are common among older adults (30-50%), often resulting in hospital admissions [6]. Multifactorial in nature, falls arise from age- and illness-related functional decline, environmental hazards, and adverse drug effects [7]. Despite extensive research, a significant knowledge gap still needs to be addressed regarding falls among older adults in Saudi Arabia.

With a population of 34 million, Saudi Arabia is undergoing a substantial demographic shift marked by an expanding aging population [5]. In 2020, over two million individuals aged 60 and older comprised more than 6% of the population, with projections indicating that this number will exceed 11 million by 2050, accounting for 24% of the population [8]. Given the country's retirement age of 60 Hijri years (approximately 58 Gregorian years), understanding the epidemiology and disability burden of falls among older Saudi adults is crucial for addressing associated challenges effectively.

Existing research on falls among older adults in Saudi Arabia is limited, yet studies suggest a considerable burden in the Middle Eastern region [9-12]. This highlights the need for further investigation to inform interventions that prevent falls among the older population in the area [13].

This study seeks to fill this knowledge gap by answering how the epidemiology and disability burden of falls among the geriatric population in Saudi Arabia (+55 years) changed over time by analyzing the Global Burden of Disease (GBD) Study data from 1990 to 2019, with two specific aims:

(1) To investigate trends in incidence and prevalence rates of falls among older adults in Saudi Arabia from 1990 to 2019, examining the changes in numbers and rates for both genders.

(2) To assess the disability burden of falls among older adults in Saudi Arabia from 1990 to 2019, analyzing trends in disability-adjusted life years (DALYs) and years lived with disability (YLDs) [14,15] for both genders, including changes in numbers and rates.

By achieving these objectives, the study addresses the challenges posed by the aging population in Saudi Arabia. The findings have significant implications for public health interventions and policymaking, with the potential to improve health outcomes and the quality of life for older adults within the country. A thorough evaluation of trends and the disability burden will guide the development of targeted preventive strategies and facilitate the identification of high-risk subgroups. This research contributes to a more comprehensive understanding and management of falls among the geriatric population.

## Materials and methods

Study design

An observational analysis was undertaken to evaluate the incidence, prevalence, and disability burden of falls among Saudi Arabian residents aged 55 years and older from 1990 to 2019. Data were assessed at seven pivotal points: 1990, 1995, 2000, 2005, 2010, 2015, and 2019, providing a comprehensive longitudinal perspective on fall-related outcomes in this demographic.

Data source and study population

The primary data source for this study was the Global Burden of Disease (GBD) dataset. This comprehensive, annually updated dataset quantifies morbidity and mortality for various diseases and injuries across age groups, sexes, and countries [14]. The GBD dataset is compiled from multiple sources, including vital registration systems, verbal autopsies, disease registries, and scientific literature. The GBD study has published in-depth explanations of their methodology and approach, and additional information on the methods utilized to calculate incidence, mortality, YLL, YLD, and DALY estimates [15]. In this study, falls were identified using specific codes, and the injuries resulting from falls were categorized into 47 distinct groups. The GBD uses a systematic approach that accounts for various data idiosyncrasies to estimate disease burden.

Data analysis

Incidence and Prevalence Analysis

The study quantified the incidence and prevalence of falls among individuals aged 55 and over in Saudi Arabia at seven distinct time points between 1990 and 2019. Incidence was defined as the rate of new fall cases occurring between intervals between the time points. In contrast, prevalence was measured as the total number of individuals experiencing falls at each time point, representing the existing burden of the condition within the population. The percentage changes in incidence and prevalence rates were computed relative to the 1990 data, serving as the reference year for longitudinal comparison.

Disability Burden Analysis

The disability burden of falls in Saudi Arabians aged 55 years and older was measured using disability-adjusted life years (DALYs) and years lived with disability (YLDs) at predetermined intervals between 1990 and 2019. DALYs represent the sum of years of life lost (YLLs) due to premature mortality and YLDs, reflecting the total burden of disease both from mortality and morbidity. Baseline comparisons for evaluating changes in DALY and YLD rates were anchored to 1990 figures.

Ethical considerations

This study utilized de-identified secondary data from the publicly available GBD study database. Ethical approval was not required for this specific analysis. The research adheres to the highest standards of research integrity and compliance with ethical norms.

Temporal trends analysis

Temporal trends for the incidence, prevalence, DALYs, and YLDs from falls among Saudi Arabian individuals aged 55 and over were examined for the period of 1990-2019. These trends were stratified by gender and visually represented to facilitate a clear understanding of the changes across the three-decade span.

Modeling approach used in GBD

The GBD study uses a Bayesian hierarchical modeling approach to estimate the measures of interest [16]. The models fit the data using DisMod-MR 2.1, a meta-regression tool incorporating covariates, data quality adjustments, and spatial-temporal smoothing [16]. The results are presented as rates per 100,000 population and 95% uncertainty intervals (UIs). Retrospective analysis using secondary data may be subject to data limitations and potential sources of bias [17]. The generalizability of the findings may be influenced by the assumptions and methodologies employed in the GBD study [16]. Efforts were made to address these limitations and provide a robust analysis of fall incidence, prevalence, and disability burden among the geriatric population in Saudi Arabia.

## Results

The study's findings are presented alongside each estimate's 95% uncertainty interval (UI), which indicates a range of values that is likely to contain the actual number of incidence and prevalence cases, as well as the true rates of DALYs and YLDs, with a 95% probability of accuracy. Using a 95%, UI is standard practice in epidemiological research, as it accounts for the variability and possible inaccuracies in large datasets. It provides a statistical assurance of the reliability of the estimates, thus enabling more informed and cautious interpretation, particularly in the context of public health.

Incidence and prevalence of falls

In 2019 (as shown in Table 1), the incidence of falls in older Saudi Arabian males was recorded at 86,038.28, with a 95% UI of 73,796.34 to 99,566.8. For females, the incidence was 45,317.56 with a 95% UI of 39,494.38 to 52,106.65. The data show an increase in the incidence rates since 1990, with a surge of 69% for males and an increase of 30% for females in the incidence rates of falls over this period.

**Table 1:** Incidence and prevalence number and rates of falls among older adults in Saudi Arabia (+55 years) by gender and changes for 1990 and 2019

		Incidence, 1990 (95% UI)	Incidence, 2019 (95% UI)	Change (%)	Prevalence, 1990 (95% UI)	Prevalence, 2019 (95% UI)	Change (%)
Number	Male	16276.42 (13994.12, 19096.49)	86038.28 (73796.34, 99566.8)	429	153420.5 (133031.6, 179628.2)	751311.7 (648879, 886857.6)	390
	Female	11877.33 (10382.13, 13531.53)	45317.56 (39494.38, 52106.65)	282	62666.07 (55678.31, 71578.68)	232359.4 (202489.5, 269986.6)	271
	Both	28153.75 (24597.56, 32434.49)	131355.8 (113831.5, 151049.6)	367	216086.6 (188491.9, 250885.3)	983671.1 (852786, 1156452)	355
Rate (per 100000)	Male	3110.8 (2674.6, 3649.78)	5271.159 (4521.153, 6099.987)	69	29322.21 (25425.4, 34331.09)	46029.32 (39753.75, 54333.57)	57
	Female	3215.419 (2810.64, 3663.242)	4172.095 (3635.992, 4797.122)	30	16964.89 (15073.17, 19377.7)	21391.83 (18641.9, 24855.92)	26
	Both	3154.094 (2755.69, 3633.67)	4832.008 (4187.363, 5556.454)	53	24208.41 (21116.94, 28106.94)	36184.96 (31370.27, 42540.81)	49

The prevalence of falls among older males in Saudi Arabia for the year 2019 was estimated at 751,311.7 (95% UI: 648,879-886,857.6), and for females, it was approximately 232,359.4 (95% UI: 202,489.5-269,986.6). These data indicate the total count of individuals who had experienced falls within the year. Compared to the 1990 rates, there has been an increase of 57% for males and 26% for females in the prevalence rates of falls.

Furthermore, the incidence rate for every 100,000 men aged 55 and above was 5,271.159 new cases of falls in 2019. For women in the same age group, the incidence rate was 4,172.095 per 100,000. The prevalence rates per 100,000 population stood at 46,029.32 for males and 21,391.83 for females.

Disability burden of falls

The burden of falls was assessed by using measures such as DALYs and YLDs. DALYs take into account the years lost due to premature death and years spent living with a disability, whereas YLDs only consider the years lived with a disability. These rates are calculated by dividing the relevant measure by the number of people at risk in a particular population.

The DALY numbers are shown in Table 2. Falls resulted in a loss of 49,028 DALYs in older men (aged 55 and older) in 2019 and 14,788 DALYs in older women in the same year (2019). This means that falls cause significant disability and premature death in these age groups. The number of DALYs caused by falls has increased since 1990. In 1990, falls resulted in a loss of 16,794 DALYs in older men and 6,669 DALYs in older women. In 2019, the rate of DALYs for falls in males was 1,921 per 100,000, while for females, it was 875 per 100,000. This implies that out of every 100,000 older men and women in Saudi Arabia, 1,921 and 875 suffered from a disability or untimely death due to falls. The DALYs rate for falls has increased since 1990. In 1990, the DALY rate for falls in older males was 1,854 per 100,000 males. Between 1990 and 2019, the DALY rate for females decreased by 10% from 968 to 875 DALYs per 100,000 females. This decrease could be due to various reasons, such as reducing the severity of falls among women or improving the quality of care for those who experience falls. As a result, there has been a decline in the number of years lived with disability.

**Table 2:** Disability burden of falls among older adults in Saudi Arabia (+55 years) by gender: DALY and YLD number, rates, and changes for 1990 and 2019

		DALYs, 1990 (95% UI)	DALYs, 2019 (95% UI)	Change (%)	YLDs, 1990 (95% UI)	YLDs, 2019 (95% UI)	Change (%)
Number	Male	16794.13 (12176.56, 22091.31)	49028.88 (36596.97, 62715)	192	6558.428 (4682.13, 8937.065)	28135.36 (19392.22, 39592.19)	329
	Female	6669.958 (4927.688, 8460.885)	14788.5 (11365.7, 19004.64)	122	3060.278 (2226.031, 4082.946)	9743.854 (6840.576, 13442.14)	218
	Both	23464.09 (17735.36, 30161.18)	63817.37 (48520.73, 81781.86)	172	9618.706 (6941.37, 13048.35)	37879.21 (26292.63, 53003.91)	294
Rate (per 100,000)	Male	1854.499 (1344.602, 2439.442)	1921.658 (1434.397, 2458.078)	4	1253.467 (894.863, 1708.079)	1723.721 (1188.069, 2425.626)	38
	Female	968.5309 (715.5395, 1228.588)	875.4675 (672.8409, 1125.06)	-10	828.4752 (602.6287, 1105.331)	897.0536 (629.7676, 1237.53)	8
	Both	1471.789 (1112.453, 1891.866)	1504.916 (1144.196, 1928.547)	2	1077.594 (777.6489, 1461.82)	1393.411 (967.191, 1949.782)	29

The YLD numbers in Table 2 show that the number of years lived with disability (YLDs) due to falls in older adults in Saudi Arabia has increased since 1990. In 2019, there were 28,135 YLDs for older males and 9,743 YLDs for older females. In 1990, these numbers were 6,558 YLDs for older males and 3,060 YLDs for older females. In 2019, the YLD rate for falls in older males was 1,723 per 100,000 males in Saudi Arabia, indicating that 1,723 men aged 55 and above were experiencing some disability due to a fall. Similarly, the YLD rate for falls in older females was 897 per 100,000 females, meaning that 897 women aged 55 and above were living with some disability due to a fall. Since 1990, the YLD rate for falls has risen. In that year, older males had a YLD rate of 1,253 per 100,000 males, while older females had a YLD rate of 828 per 100,000 females.

Trends in incidence and prevalence rates

**Figure 1:**
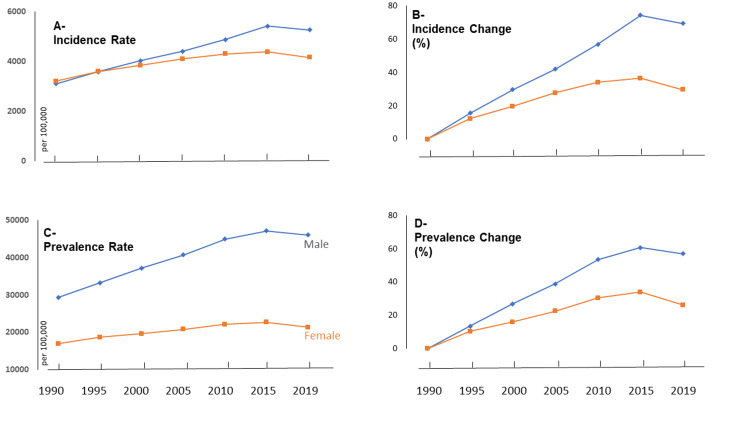
Trends of incidence and prevalence rates of falls and changes among older adults in Saudi Arabia (1990-2019)

Figure 1A displays the growing incidence rates of falls among older adults in Saudi Arabia from 1990 to 2019, with separate lines for males and females. In 2019, the incidence rate reached 5,271 per 100,000 males and 4,172 per 100,000 females.

Figure 1B shows the percentage change in incidence rates since 1990, with males experiencing a 69.45% increase and females a 29.75% increase, highlighting gender-specific variations. Figure 1C presents the prevalence rates of falls among older adults, revealing an increase from 1990 to 2019 for both genders: males rose from 29,322 to 46,029 per 100,000 and females from 16,964 to 21,392 per 100,000. Figure 1D illustrates the percentage change in prevalence rates since 1990, with a 56.98% increase for males and a 26.09% increase for females, emphasizing gender differences in fall burden among older adults in Saudi Arabia.

These figures reveal that, from 1990 to 2019, both the incidence and prevalence rates of falls among older adults in Saudi Arabia have increased for both genders. The increase has been more pronounced for males.

Trends in disability burden rates

**Figure 2:**
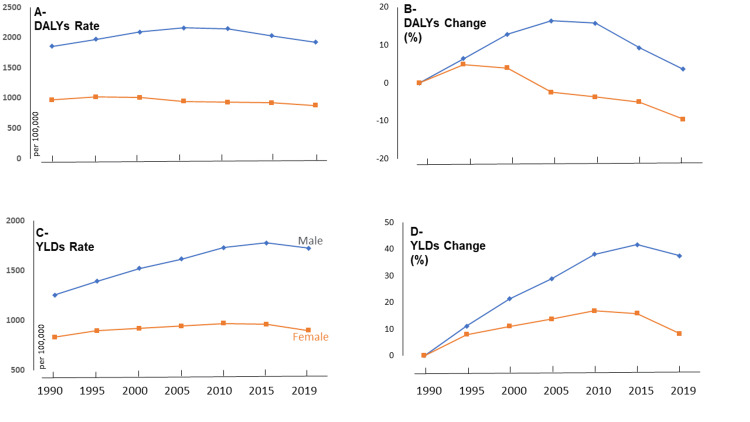
Trends of disability burden rates of falls and changes among older adults in Saudi Arabia (1990-2019)

The data presented in Figures 2A, 2B, 2C, 2D highlight the changes in disability burden rates due to falls among older adults in Saudi Arabia over 30 years (1990-2019), taking into account both DALYs and YLDs. The data reveal different trends for males and females. The DALY rates decreased by 10% for females between 1990 and 2019, while the YLD rates increased by 8.28%. This suggests that, for females, the overall health loss due to falls has lessened slightly over time, but the number of years lived with a disability has increased. For males, the DALY rates increased by 3.62%, and the YLD rates increased by 37.52% during the same period. This indicates that the overall health loss and years lived with disability due to falls have risen for older males in Saudi Arabia.

## Discussion

This longitudinal analysis of the Global Burden of Disease Study data is the first that provides valuable insights into the changing incidence, prevalence, and disability burden of falls among the geriatric population in Saudi Arabia over 30 years. The findings indicate a substantial increase in the country's new and existing rates of falls among older adults. Furthermore, the disability burden rates displayed gender-specific trends, with an increase for males and a slight decrease for females in DALYs but an overall rise in YLDs for both genders. This discussion will focus on interpreting these findings, comparing them with other studies, exploring their implications for policy and practice, and providing recommendations for future research and directions.

The increased incidence and prevalence of falls among older adults in Saudi Arabia during the study period may be attributed to various factors, including prolonged lifespan, heightened awareness and reporting of falls, the prevalence of risk factors such as chronic diseases, and conceivably environmental and lifestyle modifications that impact this age group. One potential explanation is the country's rapidly aging population [12,18]. As the proportion of older adults grows, the number of individuals at risk for falls also rises. Additionally, the increased prevalence of chronic conditions and multimorbidity among older adults [19,20] may contribute to an elevated risk of falls due to their impact on physical function and mobility. The gender-specific trends in the disability burden of falls, particularly the slight increase in DALYs for males and the slight decrease for females, may be influenced by various factors. Generally, males are more likely to engage in risky behaviors that may lead to falls [21]. Additionally, differences in physical activity levels, muscle strength, and balance between males and females may contribute to variations in the risk of falls and related disability [22-24]. The observed increase in YLDs for both genders may be partially attributed to improvements in healthcare and access to rehabilitation services, reducing the severity of fall-related disabilities.

The findings of this study are consistent with previous research highlighting the increasing burden of falls among older adults globally [25-26]. Falls are more common in higher-income countries among older adults, emphasizing the importance of global prevention efforts [27]. This may partly explain the substantial fall increase followed by Saudi Arabia, a high-income country experiencing rapid socioeconomic development [18]. Furthermore, these findings are aligned with previous studies that have reported gender-specific differences in fall risk and disability burden [3]. Similar to these results, a study conducted in the United States by Orces et al. [26] found an increase in the prevalence of falls and fall-related injuries among older adults. However, the study also reported a decline in fall-related mortality among both genders, suggesting improvements in managing fall-related injuries [27] and suggesting the need for a comparable local program to address this disabling condition nationally feasibly.

The increasing burden of falls among older adults in Saudi Arabia underscores the need for targeted prevention and management strategies. Policymakers, healthcare providers, and community organizations must collaborate to develop and implement comprehensive and evidence-based approaches to reduce falls and improve the well-being of older adults. These may include multifactorial fall risk assessments, home safety evaluations, medication reviews, vision and hearing checks, and exercise programs to improve balance, strength, and mobility [20,28]. Moreover, these findings highlight the importance of addressing gender-specific risk factors and tailoring interventions to meet the unique needs of males and females [29]. For example, male-focused interventions may emphasize the importance of risk reduction and adopting safer behaviors. In contrast, female-focused interventions may prioritize strategies to improve bone health and prevent osteoporosis, a common risk factor for falls among older women [30].

It would be beneficial for future research to delve into the root causes that lead to the growing issue of falls among older individuals in Saudi Arabia. Identifying any factors that can be modified to reduce this risk would be particularly useful. To develop effective strategies for preventing falls and related disability outcomes, conducting clinical trials with long follow-ups that analyze the impact of specific interventions considering the local Saudi culture is essential. Moreover, research should explore the cultural, social, and environmental factors that may influence fall risk and prevention behaviors among older adults in Saudi Arabia. This may include examining the role of caregiver support, religious practices such as walking daily to worship places frequently, access to healthcare services, and the built environment in determining fall risk and the effectiveness of prevention strategies. Such research can help guide the development of culturally appropriate interventions and inform policy decisions regarding allocating resources and prioritizing fall prevention initiatives.

When interpreting the findings derived from the GBD study, it is essential to acknowledge and address various limitations. These include potential limitations related to the quality and availability of the data used in the study, assumptions made during the modeling process, inherent uncertainties associated with estimates, possible temporal lag in the data, challenges related to data aggregation and comparability, the absence of causal inference due to the observational nature of the study design, and ethical considerations concerning the use of DALYs as a metric. By recognizing these limitations, researchers and stakeholders can better contextualize and understand the implications of the study's findings [15]. Moreover, it is important to acknowledge certain limitations regarding the estimation methods employed in the GBD study, particularly in the specific context of Saudi Arabia, characterized by rapid socioeconomic development and evolving demographics. Additionally, the ecological nature of the study design restricts the ability to identify individual-level risk factors for falls and associated disability outcomes, potentially affecting the generalizability of the findings to diverse populations or settings. Furthermore, it should be noted that the study's data collection concluded in 2019, which may not capture more recent trends in the incidence, prevalence, and disability burden of falls among older adults in Saudi Arabia. Continued surveillance and monitoring of falls and related outcomes are essential to guide policy and practice in the country.

The strength of this study lies in its comprehensive analysis using his study utilized the latest available data from the GBD dataset, which provides a robust and representative assessment of the changing incidence, prevalence, and disability burden rates of falls among older adults in Saudi Arabia from 1990 to 2019. By examining long-term trends and gender-specific patterns, this study offers valuable insights into the growing public health concern posed by falls in the geriatric population of Saudi Arabia. The utilization of high-quality data and a wide-ranging timeframe enhances the reliability and validity of the findings, making them a valuable resource for future research and informing evidence-based interventions.

## Conclusions

The epidemiological analysis of falls in Saudi Arabia's older adult population from 1990 to 2019 has revealed significant trends and gender-based differences. The data show that both males and females have experienced increased incidence and prevalence rates of falls, with males experiencing a steeper increase. The increasing number of falls among older men poses a significant public health challenge. This trend has led to an increase in disability from falls, which negatively affects the quality of life and functional independence of older adults. The percentage changes in fall-related injuries also show a disparity between genders. Therefore, it is important to continuously monitor fall-related injuries and consider gender when conducting epidemiological research on falls.
